# Identifying Natural syNergist from *Pongamia pinnata* Using High-Speed Counter-Current Chromatography Combined with Isobolographic Analysis

**DOI:** 10.3390/molecules22030397

**Published:** 2017-03-03

**Authors:** Hao Yin, Yubai Wei, Rouwen Chen, Si Zhang, Lijuan Long, Hang Yin, Xinpeng Tian, Weihong He

**Affiliations:** 1 Key Laboratory of Marine Bio-resources and Ecology, South China Sea Institute of Oceanology, Chinese Academy of Science, Guangzhou 510301, China; ybwei@scsio.ac.cn (Y.W.); chenrouwen15@mails.ucas.ac.cn (R.C.); longlj@scsio.ac.cn (L.L.); xinpengtian@scsio.ac.cn (X.T.); weihonghe@scsio.ac.cn (W.H.); 2 Guangdong Key Laboratory of Marine Material Medica, South China Sea Institute of Oceanology, Chinese Academy of Sciences, Guangzhou 510301, China; 3 College of Life Science, University of Chinese Academy of Sciences, Beijing 100049, China; 4 College of Pharmacy, Guiyang, Guizhou 550001, China; yhem@21cn.com

**Keywords:** high-speed counter-current chromatography (HSCCC), *Pongamiapinnata*, natural synergists, isobolographic analysis, subtracted residue, chemical subtraction

## Abstract

For identifying the synergistic compounds from *Pongamia pinnata*, an approach based on high-speed counter-current chromatography (HSCCC) combined with isobolographic analysis was designed to detect the synergistic effects in the complex mixture. In the approach, the complex mixture was considered as the combination of two individual samples for isobolographic analysis: the target compound and the mixture with complete removal of the target compound (subtracted residue). The two samples were prepared by HSCCC, and were used for the calculation of the expected effect of their combination. Using this approach, three compounds representing the major peaks in the HPLC of the brine shrimp toxic extract from *P. pinnata* (brine shrimp lethality test (BST) LC_50_ 36.5 μg/mL), pinnatin (**1**), 3,7-Dimethoxy-3′,4′-methylenedioxy flavone (**2**), and karanjin (**3**), were prepared from the extract, and were tested for their synergistic potency by BST. The two-phase solvent system containing *n*-hexane-ethyl acetate–MeOH–water (14:7:10:10, *v*/*v*/*v*/*v*) was selected for the one-step HSCCC separation according to the partition coefficient values (*K*). The extract was separated into seven fractions (Fr1–7) by HSCCC with a total mass recovery of 96.3%. Fr2, 4, and 6 were the peak fractions corresponding to compounds **3**, **2**, and **1**, respectively. The purities and recoveries of the target compounds after the chromatographic analysis were 95.9%–97.5% and 92.2%–96.1%, respectively. The subtracted residue of each target compound was performed by recombining all HSCCC fractions except the fraction containing the target compound. Isobolographic analysis disclosed a significant synergistic effect between karanjin and its subtracted residue (potency ratio of 0.47), which gave clear evidence that the toxicity of the extract results from synergistic interactions, and karanjin was one of the synergists participating in the interaction. The other two compounds were excluded from the synergism because these two compounds showed additive effects with their subtracted residues. Karanjin was the first synergistic compound identified from *P. pinnata*.

## 1. Introduction

The mangrove plant *Pongamia pinnata* (Leguminosae) is a fast-growing glabrous deciduous tree that is widely distributed in Southeast Asia to the sandy coast of the Pacific Ocean. The mangrove plant is well known as a plant pesticide [1,2]. Some evidence suggested the existence of synergy in the plant [3], but the synergistic ingredients remain unknown. As a part of our study of the chemical defenses of mangrove plants distributed in the intertidal zone of southern China, the synergy of toxicity among secondary metabolites in *P. pinnata* was investigated using the brine shrimp lethality test (BST).

Synergy is defined as the interaction among compounds that produces a greater effect than expected based on individual activities [4]. Isobolographic analysis, an intuitive method used in pharmacology for analyzing the effects of combinations of drugs, was then introduced to detect and rigorously quantify interactions among natural products. For example, two brominated cyclic dipeptides released by the coldwater marine sponge *Geodia barretti* acting in synergy have been detected based on this method [5]. Although progress has been made ever since the introduction of isobolographic analysis, we suggest that some synergisms have been relatively ignored because the methods for ensuring that all synergistic components necessary to reproduce the synergy are included in the combination of individual samples were rarely even mentioned in the literature. For instance, the pure compounds isolated from the extract of an organism constitute a common class of individual samples for isobolographic analysis [6,7,8,9]. In practice, the number of pure compounds available from an organism is usually limited because of the enormous diversity of secondary metabolites and the tedious steps involved in the preparation of the pure compounds.

**Figure 1:**
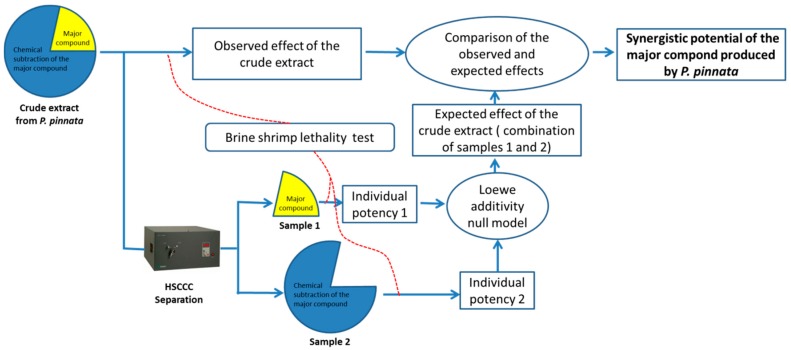
General workflow for the determination of the synergistic potential of a major compound produced by *P. pinnata.*

An approach that can ensure the integrity of synergistic components during the preparation of individual samples was developed in the present study. As illustrated in Figure 1, the complex mixture of secondary metabolites containing the target compound is considered as a combination of two individual samples: the target compound and the extract with complete removal of the target compound (subtracted residue), which is called the chemical subtraction of the target compound by Chen [10]. The two individual samples can ensure that all synergists in the starting material are included in the combination of the individual samples because the combination is exactly equal to the extract.

The compounds representing the major peaks in the HPLC chromatogram of the complex mixture, a toxic extract from *P. pinnata* (BST LC_50_ 36.5 μg/mL), were selected as the target compounds. After establishing the proper solvent system for high-speed counter-current chromatography (HSCCC) separation, the target compounds **1**–**3** and their subtracted residues were prepared from the extract. The synergistic potency of the compounds was successfully evaluated by isobolographic analysis using subtracted residue as the individual sample. The presence of synergy of toxicity in *P. pinnata* was confirmed; one of the synergists, karanjin, was obtained from the crude extract, and the other two compounds were excluded from the synergistic compounds.

Compared with the conventional method using pure compounds as individual samples for isobolographic analysis [5,6,7,8,9], the method proposed here may be more efficient because the synergistic effect of karanjin can be detected when the compounds interacting with karanjin are unknown.

## 2. Results and Discussion

### 2.1. HSCCC Separation

**Figure 2:**
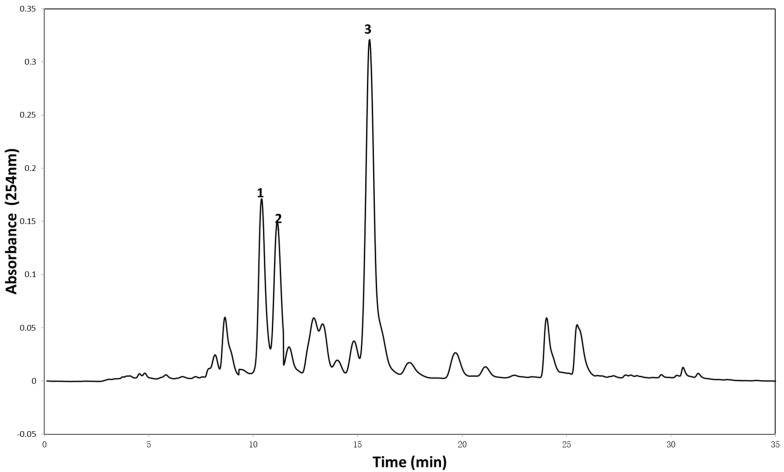
HPLC profile of the petroleum extract of *P. pinnata* and the compounds, pinnatin (**1**), 3,7-Dimethoxy-3′,4′-methylenedioxy flavone (**2**), and karanjin (**3**), corresponding to the major peaks.

As shown in Figure 2, the HPLC chromatogram of the petroleum extract of *P. pinnata* showed three major peaks corresponding to three flavonoids, which were identified as pinnatin (**1**), 3,7-Dimethoxy-3′,4′-methylenedioxy flavone (**2**), and karanjin (**3**) by comparison with published NMR data [11].

**Figure 3:**
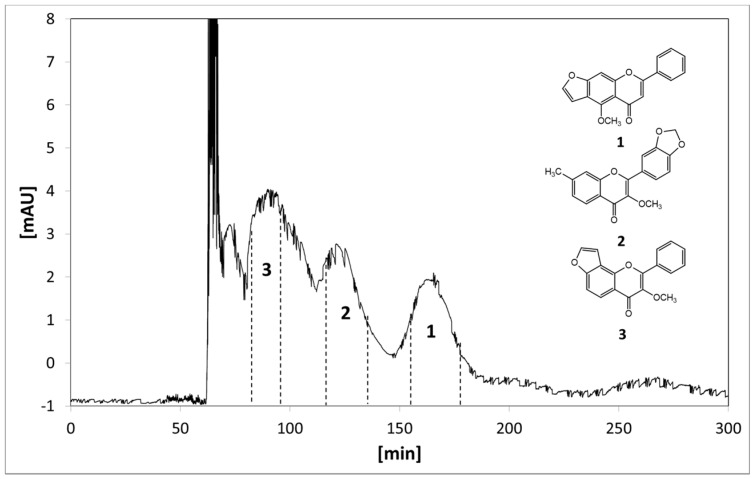
HSCCC chromatogram of the petroleum extract of bark of *P. pinnata*.

The solvent system of *n*-hexane–ethyl acetate–MeOH–water (14:7:10:10, *v*/*v*/*v*/*v*) provided suitable *K* values for all three target compounds and was finally selected for HSCCC separation. The crude extract (61.3 mg) was separated by HSCCC, and the typical HSCCC chromatogram was shown in Figure 3. 

**Figure 4:**
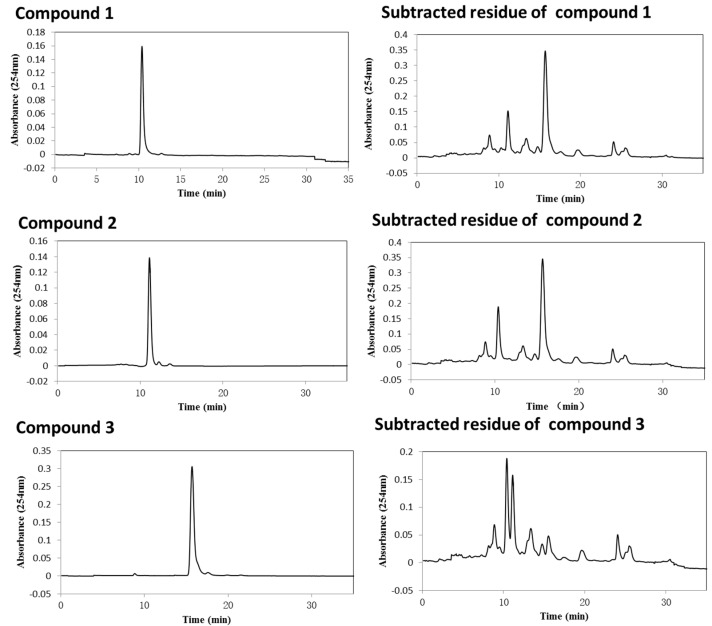
HPLC analysis of compounds **1**–**3** and their subtracted residues prepared by HSCCC.

The retention of the stationary phase was 55.3%, and the separation time was 300 min. A total of 39 fractions were collected during the entire chromatography. From 60 min after sample injection, fractions 1–32 were collected at 2.5 min intervals, and fractions 33–38 were collected according to the chromatogram. Fraction 39 was the column contents collected by pressure with nitrogen when the HSCCC centrifuge was stopped. These fractions were pooled into seven fractions (Fr1, 60–82.5 min; Fr2, 82.5–95 min; Fr3, 95–115 min; Fr4, 115–135 min; Fr5, 135–152.5 min; Fr6, 152.5–180 min; Fr7, 180–300 min and the column contents) based on their HPLC profiles, and Fr2, Fr4, and Fr6 correspond to the target compounds **3**, **2**, and **1** (Figure 4), respectively. The HPLC chromatograms of Fr1–7 showed that there is no significant chemical overlap between these fractions.

The dry weights of the fractions yielded by HSCCC are listed in Table 1. The total mass recovery was 96.3% after separating the extract using HSCCC. The recoveries and purities of the target compounds after chromatography were calculated by comparison of the peak area with those of the standards which were prepared in preliminary experiments. These fractions were directly used for NMR analyses and isobolographic analysis. The subtracted residues of the target compounds were prepared by recombining all HSCCC fractions except those containing the target compounds, and their HPLC chromatograms are listed in Figure 4.

### 2.2. Synergistic Effect between Compounds and the Subtracted Residue

**Figure 5:**
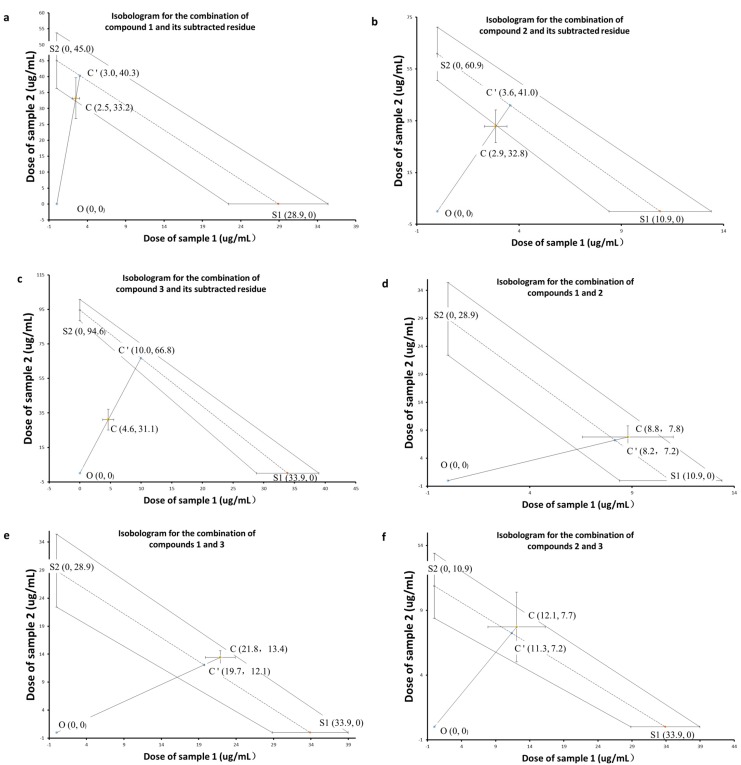
Isobolograms for the combinations of compound **1** and its subtracted residue (**a**), compound **2** and its subtracted residue (**b**), compound **3** and its subtracted residue (**c**), compounds **1** and **2** (**d**), compounds **1** and **3** (**e**), and compounds **2** and **3** (**f**).

The interaction between the compound and the subtracted residue of each sample pair was tested by using an isobolographic analysis method, and the results are presented in Table 2 and Figure 5. Analysis of the individual dose-response curves of the lethal effect against brine shrimp produced LC_50_ values for the individual samples (z_1_* and z_2_*). The LC_50_ values of the crude extract and the recombined crude extract obtained by recombining all HSCCC fractions were determined as 36.5 and 35.7 μg/mL, respectively, and the latter was used as the observed dose of the combination (Z_t_) for all three sample pairs containing subtracted residue. Each combination of equieffective pair is administered in a fixed ratio consisting of proportions p1 of the target compound (sample 1) and p2 of the subtracted residue (sample 2), and the p1 and p2 were calculated from the dry weights of the HSCCC fractions (Table 1). Each value for the expected dose (z_add_) was calculated from the values of p1, p2, z_1_* and z_2_* [12]. The data for the analysis of the synergy between any two of the three pure compounds prepared by HSCCC also are listed in Table 2 and Figure 5d–f.

As shown in Figure 5c, a significant synergistic effect of karanjin with its subtracted residue was observed. In the isobologram, the straight dashed line represents the zero-interaction line. The lines surrounding the zero-interaction isobole represent the 95% confidence interval. The LC_50_ values for compound **3** and its subtracted residue were plotted as the intercepts of the zero-interaction isobole for each axis. Point C representing the observed LC_50_ value of the combination of the two individual samples, also with the 95% confidence intervals shown, is below the zero-interaction isobole. Because the confidence intervals for the combination and the zero-interaction isobole do not overlap, one can conclude that the interaction significantly deviates from the zero-interaction line. That is, the two individual samples are more potent in combination than one would expect based on their individual effective doses. To measure the intensity of the interaction using a potency ratio, two overlapping lines, OC and OC’, are also shown radiating from the origin. The two lines represent two distances: the distance from the origin to the observed LD_50_ value coordinates of the combinations (OC), and the distance from the origin to the expected LD_50_ coordinates (OC’). This particular observed/expected distance ratio represents a potency ratio of 0.47, a measurement of the intensity of the synergism between karanjin and its subtracted residue.

Figure 5a,b show the zero interactions of compounds **1** and **2** with their subtracted residues, respectively, because the observed combination points lie along the zero-interaction lines, and the confidence intervals of the combinations overlapped with those of the zero-interaction lines. The isobolographic analysis results gave clear evidence that the toxicity of the petroleum extract of *P. pinnata* against brine shrimp results from synergy, and that karanjin was a synergist participating in the interaction. Pinnatin and 3,7-dimethoxy-3′,4′-methylenedioxy flavone were excluded from the synergists when the subtracted residues were used as individual samples to avoid the misdetection caused by the loss of synergists. None of the three combinations of the pure compounds exhibited a synergistic effect in BST (Figure 5d–f), which was compatible with the results of those isobolographic analyses using the subtracted residues as individual samples.

## 3. Materials and Methods

### 3.1. Materials and Reagents

All organic solvents used for HSCCC were of analytical grade, and purchased from Baishi Co. Ltd. (Tianjin, China). Methanol and acetonitrile used for HPLC were obtained from Merk (Darmstadt, Germany). Water was purified using a Milli-Q system (Millipore, Bedford, MA, USA).

### 3.2. Plant Collection and Extraction

*P. pinnata* was collected in April 2015 from Hainan Province, Sanya, China. A voucher sample (GKLMMM-015A) is kept in the Herbarium of the South China Sea Institute of Oceanology. Approximately 1 kg dry powdered bark (1 kg) of *P. pinnata* was extracted with EtOH (95%) three times at room temperature. After removal of the solvent by evaporation, the residue (42.2 g) was suspended in water and then successively extracted with petroleum ether, ethyl acetate and *n*-butanol. The petroleum ether extract (12.7 g) was stored at 4 °C before HSCCC separation.

### 3.3. High-Speed Counter-Current Chromatography Separation

#### 3.3.1. Analytical HPLC and Selection of the Target Compounds

A Luna 5 μm C18 100A ODS column (250 mm × 4.6 mm; Phenomenex, Torrance, CA, USA) was used for analytical HPLC, along with a 600 E Multi-solvent Delivery System and a Waters 996 Photodiode Array Detector (Waters, Milford, MA, USA). For the separation of analytes, the following gradient system was used: 0.1% formic acid in water (A) and acetonitrile (B); gradient program: 55% B, 0–13 min; 55% B to 100% B, 13–25 min; 100% B, 25–30 min; 100% B to 55% B, 30–35 min. The mobile phase was eluted at a flow rate of 1.0 mL/min and the effluent was monitored by the UV detector at 254 nm. The compounds representing the major peaks in the HPLC chromatogram of the petroleum ether fraction of crude extract were selected as the targets for this study. The sample solution was prepared by dissolving approximately 7 mg petroleum ether fraction of crude extract in 1 mL methanol, and the injection volume was 10 μL.

#### 3.3.2. Preparation of Two-Phase Solvent System and Sample Solution

The following two-phase solvent system was prepared for the HSCCC separation of target compounds from *P. pinnata*:

*n*-hexane–ethyl acetate–methanol–water (14:7:10:10, *v*/*v*/*v*/*v*)

The mixed solvent was thoroughly equilibrated in a separatory funnel at room temperature, two phases separated shortly before use. The petroleum ether extract of *P. pinnata* (61.3 mg) was dissolved in 4 mL of each phase of the solvent system for preparative HSCCC separation.

#### 3.3.3. HSCCC Separation Procedure

HSCCC was carried out using a Model TBE-300A high-speed counter-current chromatograph (Tauto Biotech Co., Ltd., Shanghai, China) containing a self-balancing three-coil centrifuge rotor equipped with three preparative multilayer coils. The internal diameter of the PTFE tubing was 2.0 mm. The radius of revolution or the distance between the holder axis and central axis of the centrifuge (R) was 9.5 cm. The *β* value varied from 0.46 at the internal terminal to 0.73 at the external terminal (*β* = r/R, where r is the distance from the coil to the holder shaft). The HSCCC system was equipped with a Model TBP-50 constant flow pump (Tauto Biotech Co., Ltd., Shanghai, China) and an LC757 UV detector with preparative flow cell (Shanghai Huixing Instrument Co. Ltd., Shanghai, China), a Model HX-1050 constant temperature controller (Beijing Detian you Technology, Beijing, China), and an N2010 chromatogram workstation (Zhejiang University, Hangzhou, China).

HSCCC separation was performed in the tail-to-head elution mode. The multilayer coiled column was first filled entirely with the lower phase of the solvent system. The upper phase was then pumped into the column at a flow rate of 2.0 mL/min while the apparatus was run in reverse mode at a revolution speed of 850 rpm. After hydrodynamic equilibrium was established as indicated by a clear mobile phase eluting at the tail outlet, the sample solution was injected through the sample port. The effluent from the head end of the column was continuously monitored with a UV detector at 254 nm. Fractions were collected according to time intervals or peaks. After the rotation and elution were stopped, the last fraction, the column contents, was collected by forcing the column contents out with pressurized nitrogen gas. The fractions were analyzed by HPLC and were then pooled according to similarities of HPLC profiles. These pooled fractions were evaporated to dryness in vacuo, dried thoroughly over P_2_O_5_, and weighed accurately for NMR analysis and isobolographic analysis.

### 3.4. Determinations of Interactions between the Compounds and Their Subtracted Residues

#### 3.4.1. Brine Shrimp Lethality Test

Artificial seawater was prepared by dissolving 38 g of sea salt (Sigma Chemical Co., Poole, UK) in 1 L of distilled water. The eggs (Advanced Hatchery Technology Inc., Salt Lake City, UT, USA) were hatched in a conical flask containing 300 mL artificial seawater. The flasks were well aerated with the aid of an air pump and kept in a water bath at 25 °C. After 48 h, the larvae (nauplii, instar II-III nauplii) were attracted to one side of the vessel with a light source and collected using a pipette. Nauplii were separated from eggs by aliquoting them three times into small beakers containing seawater. All samples and standards were dissolved and serially two-fold diluted in dimethyl sulfoxide (DMSO) and seven different concentrations were obtained. Artificial seawater (195 μL) containing 15–20 larvae was placed in each well of a 96-well microtiter plate. A sample solution (5 μL) of each concentration was transferred into the first seven wells in the appropriate column in order of ascending concentration, and the last row with seawater and DMSO only served as the drug-free control. Potassium dichromate was used as a positive control. Each sample analysis was repeated three times in each experiment. After 24 h of exposure to the samples at 25 °C, the numbers of dead and total (determined after killing by freezing) brine shrimp were determined for each well. The data were analyzed by the method proposed by Nelson [13].

#### 3.4.2. Preparation of the Subtracted Residues

The subtracted residue of each target compound was prepared separately as follow: except the fraction containing the target compound, all dried fractions produced by the HSCCC separation were re-dissolved in separately 1 mL chloroform separately. An equal volume (50 μL) of solution was removed from each fraction, and these solutions were recombined to obtain the subtracted residue of the target compound.

#### 3.4.3. Isobolographic Analysis

The interaction between two samples (sample 1 and sample 2) was determined by using the method reported by Tallarida [12], and Loewe and Muischnek [14].

## 4. Conclusions

Using the approach based on HSCCC and isobolographic analysis, the presence of synergy of toxicity in *P. pinnata* was confirmed, one of the synergists was obtained from the crude extract, and the other two compounds were excluded from the synergistic compounds.

*P. pinnata* is one of the most common botanical pesticides used against pests of economic importance, as famous as the neem (Azadirachtaindica) [15]. The oil and extract of *P. pinnata* have been evaluated and found to act as oviposition deterrents, antifeedants and larvicides against a wide range of insect pests. However, the previous investigations on *P. pinnata* have not resulted in any compounds as potent as azadirachtin from neem. The synergy between components of whole plant extracts may explain why crude extracts are more effective than isolated constituents [16]. Some evidence suggested the existence of synergy in *P. pinnata* [3], but the synergistic ingredients remained unknown. In the present study, the synergistic compound karanjin was firstly identified from the crude extract for first time, which might help in understanding the chemical defense system of the plant, and could help fully explore the potential of *P. pinnata* for sustainable integrated pest management programs.

In natural synergistic research using isobolographic analysis, the pure compounds isolated from the extract of an organism constitute a common class of individual samples for isobolographic analysis [4,5,6]. The advantage of the pure compounds is that not only evidence of synergy but also all synergistic compounds may be obtained. However, the number of available pure compounds from an organism is usually limited. The combinations of these selected compounds have a low probability of including all synergists necessary for reproducing the synergy. It is therefore reasonable to deduce that some synergistic effects have not been reproduced and detected by isobolographic analysis. Another common class of samples is the mixtures of natural products, such as chromatographic fractions. The mixture samples are more suitable for detecting synergy rather than identifying synergistic compounds. The target compound and its subtracted residue were used as individual samples for isobolographic analysis in the method proposed here, which can rigorously demonstrate synergistic or antagonistic interactions participated in by the target compound. The drawback of the method is that the synergy occurring in the crude extract should not be detected when the target compound is not synergistic. The method is extremely effective for identifying the synergistic effect of the compounds prepared from the complex mixture by HSCCC because the subtracted residue could be obtained from the chromatographic fractions without any additional separation.

## Figures and Tables

**Table 1:** Weights of fractions, purities and recoveries of the target compounds, and the total mass recovery after the HSCCC separation.

	Fr1	Fr2 (Compound 3)	Fr3	Fr4 (Compound 2)	Fr5	Fr6 (Compound 1)	Fr7	Sum
Dry weight (mg)	17.6	7.5	7.1	4.5	4.3	4.2	13.6	58.8
Purity (%)	\	96.1	\	95.9	\	97.5	\	\
Recovery (%)	\	92.2	\	94.9	\	96.1	\	96.3 ^a^

^a^ Total mass recovery.

**Table 2:** Calculated and observed BST LC_50_ and proportions of samples 1 and 2 in combination for six sample pairs prepared by HSCCC.

Combination (Sample 1:Sample 2)	p1 ^a^	p2 ^a^	z_1_*	z_2_*	Z_t_	Z_add_^c^
			BST LC_50_, μg/mL ^b^			
Compound **1**:subtracted residue	0.07	0.93	28.9 ± 6.5	45.0 ± 8.7	35.7 ± 6.7	43.3
Compound **2**:subtracted residue	0.08	0.92	10.9 ± 2.5	60.9 ± 10.3	35.7 ± 6.7	44.6
Compound **3**:subtracted residue	0.13	0.87	33.9 ± 11.1	94.6 ± 6.2	35.7 ± 6.7	76.8
Compound **1**:compound **2**	0.53	0.47	10.9 ± 2.5	28.9 ± 6.5	16.6 ± 6.9	15.4
Compound **1**:compound **3**	0.62	0.38	33.9 ± 5.1	28.9 ± 6.5	35.3 ± 3.2	31.8
Compound **2**:compound **3**	0.61	0.39	33.9 ± 5.1	10.9 ± 2.5	19.8 ± 4.2	18.5

^a^ The values p1 and p2 were the proportions of samples 1 and 2 in the combination and equal to those in the petroleum extract of *P. pinnata*; ^b^ The LC_50_ ± SD of the individual samples; ^c^ $Z_{\text{add}}=1/\left[\frac{p_{1}}{z_{1}^{*}}+\frac{p_{2}}{z_{2}^{*}}\right]$.

## Supplementary Files

Supplementary File 1
