# Supplementary material for: Identifying Natural syNergist from Pongamia pinnata Using High-Speed Counter-Current Chromatography Combined with Isobolographic Analysis
Source: Molecules. 2017 Mar 3;22(3):397. doi: 10.3390/molecules22030397 (PMC6155414; doi:10.3390/molecules22030397)
Supplement: Supplementary File 1 [file molecules-22-00397-s001.pdf]

Supplementary Materials: Identifying natural synergist from *Pongamia pinnata* using high-speed counter-current chromatography combined with isobolographic analysis

HaoYin, Yubai Wei, Rouwen Chen, Si Zhang, Lijuan Long, Hang, Yin, Xinpeng Tian, and Weihong He

NMR data of Compounds 1, 2, and 3

Compound **1**, colorless needle, ^{1}H NMR ($CDCl_3$，500MHz): δ_H 6.70 (1H, s, H-3), 7.37 (1H, d, *J*=0.7 Hz, H-8), 7.90 (2H, m, H-2′,6′), 7.51 (3H, m, H-3′,4′,5′), 7.05 (1H, dd, *J*=2.2, 0.7 Hz, H-4′′), 7.63 (1H,d,*J*=2.2, H-5′′), 4.22 (3H,s, MeO-5); ^{13}C NMR ($CDCl_3$, 125 MHz): δ_C 161.3 (C-2), 107.8 (C-3), 178.4 (C-4), 153.7 (C-5) , 112.8 (C-6), 155.8 (C-7), 95.4 (C-8), 158.0 (C-9), 117.2 (C-10), 131.6 (C-1′), 126.1 (C-2′,6′), 129.0 (C-3′,5′), 131.3 (C-4′), 105.4(C-4′′), 145.3 (C-5′′), 55.2 (MeO-5).

Compound **2**, white powders，^{1}H NMR ($CDCl_3$, 500MHz): δ_H 8.17 (1H, d, *J* =8.9 Hz, H-5), 6.98 (1H, dd, *J*=8.9, 2.3 Hz, H-6), 6.91 (1H, d, *J*=2.3 Hz, H-8), 7.64 (1H, d, *J*=1.7 Hz, H-2′),6.94 (1H, d, *J*=8.3 Hz, H-5′), 7.72 (1H, dd, J = 8.3, 1.7 Hz, H-6′), 6.08 (2H, s,-OCH_2O-), 3.91 (3H, s, OMe-3), 4.02 (3H, s, O OMe-7); ^{13}C NMR ($CDCl_3$, 125 MHz): δ_C154.7(C-2), 141.8(C-3), 174.4(C-4), 127.1(C-5), 114.3(C- 6), 164.0(C-7), 99.9(C-8), 156.8(C-9), 118.1(C-10), 124.8(C-1′), 108.4 (C-2′), 147.9(C-3′), 149.5 (C-4′), 108.6(C-5′), 123.4(C-6′), 60.2(OMe-3), 55.6(OMe-7), 101.6(OCH_2O)。

Compound **3**, colorless crystal，^{1}H NMR ($CDCl_3$, 500MHz): δ_H8.20 (1H, d, *J*=8.8 Hz, 5-H), 7.54 (1H, d, *J*=8.8Hz, 6-H), 8.16 (2H, m, 2′, 6′-H), 7.57 (3H, m, 3′, 4′, 5′ -H), 7.19 (1H, d, *J*=2.2 Hz, H-4″) ,7.77 (1H, d, *J*=2.2 Hz, 5″-H), 3.95 (3H, S, OMe -3); ^{13}C NMR ($CDCl_3$, 125 MHz): δ_C154.8(C-2), 141.8(C-3), 175.1(C-4), 121.4 (C-5), 109..5(C-6), 157.7 (C-7), 116.6 (C-8), 149.6 (C-9), 119.3(C-10), 130.6(C-1′), 127.9(C- 2′, 6′), 128.3 (C-3′, 5′), 130.3 (C-4′), 103.8 (C-4′′), 145.5 (C-5′′), 59.8 (OMe-3).

Optimization of HSCCC condition

The HPLC chromatogram of the petroleum extract of *P. pinnata* showed three major peaks corresponding to compounds **1**-**3** (Figure 2). An HSCCC method was developed for the preparative separation of these compounds and their chemical subtraction from the crude extract. Successful separation by HSCCC depends largely upon the selection of a suitable two-phase solvent system, which provides an ideal range of the partition coefficient (*K*) for the targeted compounds, and the suitable K values for HSCCC are $0.5 \le K \le 2.0$. As proposed by Ito, we searched systematically for a suitable solvent system in a set of two-phase solvent systems containing n-hexane-ethyl acetate-MeOH-water. Flavonoids are known to be the major secondary metabolites in *P. pinnata*, and the *n*-hexane-ethyl acetate-MeOH-water solvent systems were previously used for successful separation of flavonoids. The *K* values of the target compounds in these two-phase solvent systems were tested. As shown in Table S1, *n*-hexane-

ethyl acetate-MeOH-water (6:4:5:5, v/v/v/v) provided K values slightly off from the proper range. The *K* values were adjusted by modifying the volume ratio, adding NH_4OH or acetic acid, respectively. The solvent system of n-hexane-ethyl acetate-MeOH-water (14:7:10:10, v/v/v/v) provided suitable *K* values for all three target compounds and was finally selected for HSCCC separation.

Table S1. The *K* (partition coefficient) values of compounds **1**-**3** in different solvent systems [1]

Solvent system (volume ratio)	Compound **1**	Compound **2**	Compound **3**
n-Hexane-Ethyl acetate-*Me*OH -water (3:5:3:5, v/v/v/v)	6.55	10.42	15.06
n-Hexane-Ethyl acetate-*Me*OH -water (4:5:4:5, v/v/v/v)	3.45	5.48	9.62
n-Hexane-Ethyl acetate-*Me*OH -water (1:1:1:1, v/v/v/v)	1.58	2.86	4.65
n-Hexane-Ethyl acetate-*Me*OH -water (6:4:5:5, v/v/v/v)	0.64	1.16	2.12
n-Hexane-Ethyl acetate-*Me*OH -water (7:3:5:5, v/v/v/v)	0.33	0.56	1.55
n-Hexane-Ethyl acetate-*Me*OH-water(14:7:10:10,v/v/v/v)	0.52	0.93	1.96
n-Hexane-Ethyl acetate-*Me*OH-0.5% AcOH (6:4:5:5, v/v/v/v)	0.78	1.21	2.15
n-Hexane-Ethyl acetate-*Me*OH-0.5%NH_4OH (6:4:5:5,v/v/v/v)	0.73	1.18	2.48

[1]Experimental procedure: for each solvent system, approximately 3 mg of extract was weighed in a 10 mL test tube into which 1 mL of each phase of the pre-equilibrated solvent system was added. The test tube was capped and shaken vigorously for 1 min and allowed to stand until it separated completely. An aliquot of 100 μL was taken from each layer and evaporated to dryness in vacuo. The residue was dissolved in 100μL acetonitrile and analyzed by HPLC to determine the partition coefficient (*K*) of compounds **1, 2** and **3**. Each *K* value was expressed as the peak area of the target compound in the upper phase divided by the peak area of the target compound in the lower phase.

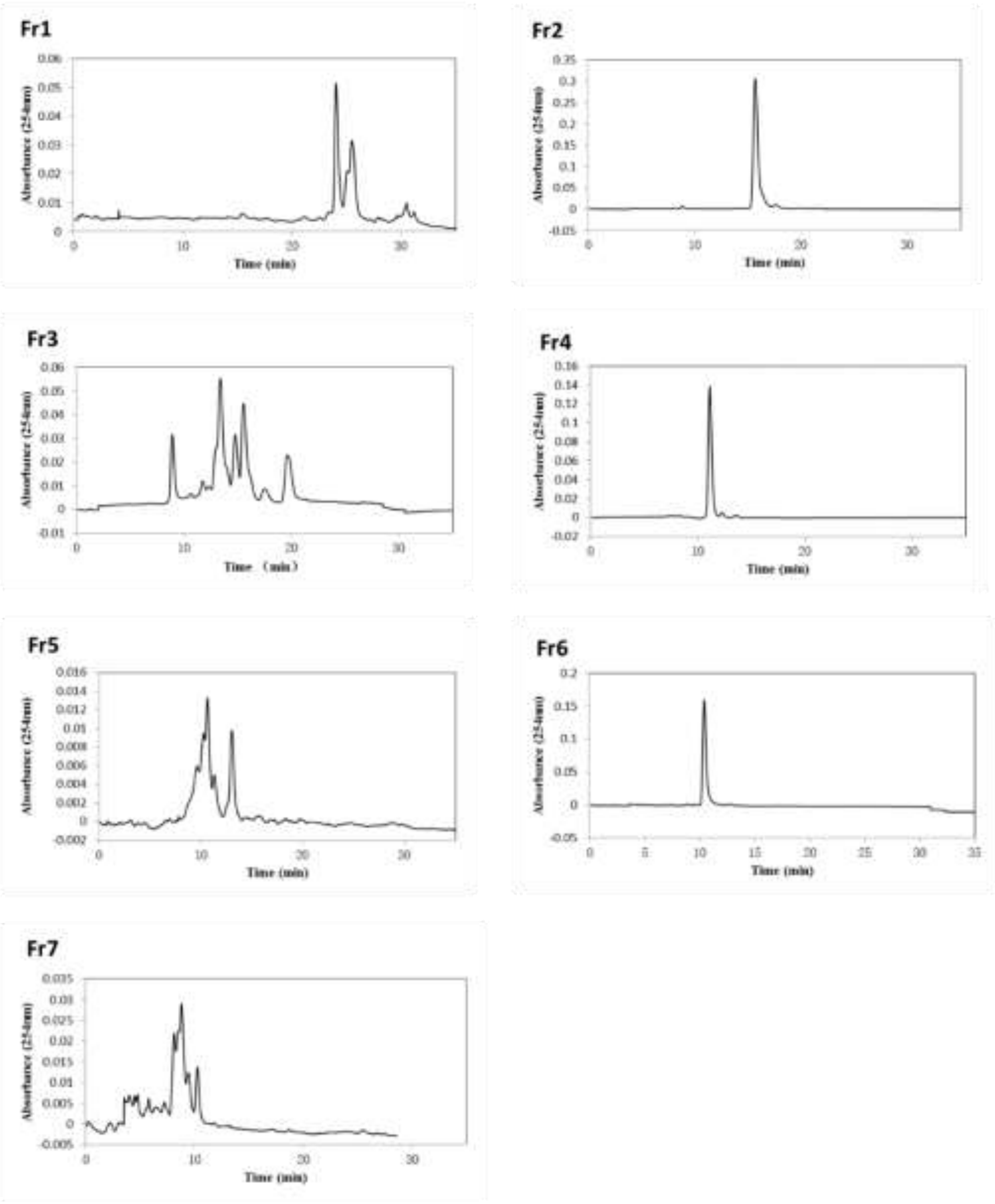

Figure S1. HPLC analysis of fractions 1~7 from the HSCCC separation
